# Does birth season correlate with childhood stunting? An input for astrological nutrition

**DOI:** 10.1186/s12887-022-03343-w

**Published:** 2022-05-24

**Authors:** Melese Linger Endalifer, Gedefaw Diress, Bedilu Linger Endalifer, Birhanu Wagaye, Hunegnaw Almaw

**Affiliations:** 1 Department of Human Nutrition, College of Health Science, Debre Markos University, Debre Markos, Ethiopia; 2 Department of Public Health, College of Health Science, Debre Markos University, Debre Markos, Ethiopia; 3 Department of Pharmacy, College of Medicine and Health Science, Wollo University, Dessie, Ethiopia; 4 Department of Public Health, College of Medicine and Health Science, Wollo University, Dessie, Ethiopia; 5 Department of Public Health Nutrition and Dietetics, College of Medcine and Health Science, Bahir Dar University, Bahir Dar, Ethiopia

**Keywords:** Season of birth, Spring, Stunting, Ethiopia

## Abstract

**Introduction:**

Chronic malnutrition is highly prevalent in Sub-Saharan Africa and a severe public health problem in Ethiopia.At country level in the past three decades,the prevalence of stunting is above 40%.Different researchs and intervention were implemented in the past;but the progresss is non-remarkable.Despite; the effect of birth season on childhood chronic malnutrition was not studied yet in Ethiopia.

**Methods:**

This research was extracted from the 2016 demographic health survey of Ethiopia. The data was collected based on national and international scientific protocols. A total of 645 enumeration areas were selected for the national survey.The surevey uses two stage stratified sampling technique to gather data from the sampling unit. After excluding non eligible children a total of 8855 participants were included for final analysis.Height and weight were measured based on the standards nutritional assessment procedure.SPSS version 20 was used to analyze the data.Descriptive statistics were used to present the data. Binary and multivariable logistic regression models were regressed to identify the potential predictors.A *p*-value of less than 0.05 with 95% CI were used to declare an association.

**Result:**

The prevalence of stunting in Ethiopia was 38.7% (95% CI: 36.8, 40.6). Season of birth had a significant association with stunting. The odds of being stunted among children born in the spring season were decreased by 16% as compared to children born in the winter season.

**Conclusion:**

Children born in the spring season were less likely to be stunted (the so called October effect). The clear scientific relation between the season of birth and child anthropometric indicator is not well understood. Nutritional interventions and policies are better to consider the birth season of the child.

## Background

Malnutrition is a global public health problem that affects the entire population. In the twenty-first century, the rates of under-nutrition and over-nutrition increased unpredictably. Unexpectedly developing countries suffered from a double burden of malnutrition the so-called “DBM” at an individual, household, and community level [1, 2].

Undernutrition is a major public health problem among adolescents,children under-five years, and pregnant and lactating women in developing countries due to the vicious pattern of the problem. Majorly the problem is highly prevalent in Sub-Saharan Africa [3–6].

Stunting is a form of chronic malnutrition in under-five children which is expressed in percent of median, Z-score, and percentiles. Based on the World Health Organization (WHO) cut-off value stunting is classified when the Z-score value is less than -2 [7].

Globally;21.3% of children under five years were stunted [3], of this 30% of them were in Sub-Saharan Africa.There is a little bit epidemiological reduction in the region [8, 9]. In the last two decades; there was observable stunting reduction in Ethiopia [10, 11]. Based on 2016, Ethiopia Demographic Health Survey (EDHS) report the prevalence of stunting was 38% and in the northern regions (highland areas) of Ethiopia (Tigray, Afar, and Amhara) stunting prevalence is above 40% which is greater than the national level [11].

The consequence of stunting is beyond the child's health. It had social, economic, political, and health crises. Nationally Ethiopia costs about 16.5% of Gross Domestic Product per annum due to malnutrition [12]. Some of the health consequences of stunting are decreased intelligence ability and secondary malnutrition (late adulthood metabolic syndrome) [13].

Generally, the determinant factors and causes of stunting are interrelated and complex. It is a known fact that a bunch of researchers identified maternal, health-related, nutritional and sociodemographic factors [3–5, 8, 9, 13–49]. Even though a lot of research was conducted before in Ethiopia and worldwide the determinant factors explored and the methodology were almost similar. Surely, in Ethiopia, the effect of birth season on childhood malnutrition was not studied before. The government and different stakeholders put their effort to reduce malnutrition; although the progress is not satisfactory [50]. There is low energy and nutrient intake in the summer season due to temporal food insecurity (hunger) in Ethiopia [51].To know the effect of season on the nutritional status of children the current type of study is fundamental.

As a result, we intend to assess the effect of the season of birth on stunting in Ethiopia nationally.

## Materials and methods

### Study setting and design

**Fig. 1:**
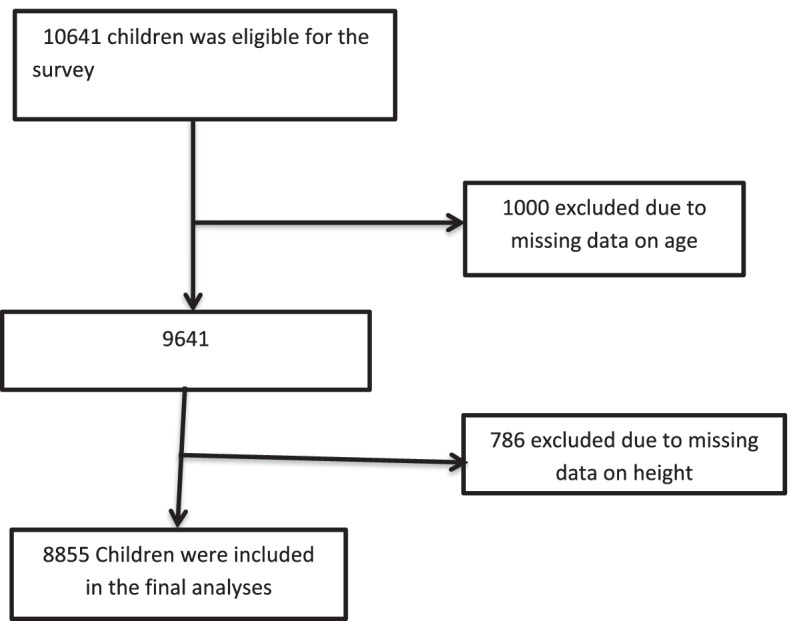
The flow diagram of the study participant’s selection

The 2016 EDHS was designed to provide up-to-date estimates of key demographic and health indicators in Ethiopia. The data was collected from nine regions and two city administrations every five years.A detailed description of the study design and methodology the 2016 is found elsewhere [52].In brief; a stratified two-stage random sampling design was used to collect data from a nationally representative sample. In the first stage, a total of 645 Enumeration Areas (EA) (202 in urban areas and 443 in rural areas) were selected with probability proportional to EA size and with independent selection in each sampling stratum. In the second stage, a fixed number of 28 households per cluster were selected with an equal probability of systematic selection from the newly created household listing. A total of 10,752 children under the age of 5 years were eligible for length/height and weight measurements in the 2016 EDHS, who are born in the last 5 years before the survey.Weight measurements were obtained using light weight SECA scales with a digital screen designed and manufactured under the guidance of UNICEF. Height measurements were carried out using a Shorr measuring board. Children younger than 24 months were measured for height while lying down position, and older children were measured in standing position. Wealth index of households are given scores based on the number and kinds of consumer goods they own, ranging from a television to a bicycle or car, in addition to housing characteristics such as source of drinking water, toilet facilities, and flooring materials. These scores are derived using principal component analysis.We excluded children with missing data on the question related to the outcomes of interest and other covariates adjusted in the multivariable model. We include a total of 8855 children under-five years for finaly analyses (Fig. 1) [52].

### Outcome of interest

The outcome of interest was stunting which was assessed by measuring the height/length and age of the children. The outcome variable was categorized as stunted and non-stunted. Stunting is classified based on WHO Z-Score value; children having a Z-Value < -2 are considered as stunted and all children having a Z-Value ≥ -2 was categorized as non-stunted [52].

### Exposure variable

Season of birth classification was taken from Ethiopian Metrological Data. In Ethiopia context, there are four major seasons; spring, winter, autumn, and summer. In each season three months were incorporated. In the spring (September, October, and November), winter (December, January, and February), autumn (March, April, and May), and summer(June, July, and August). Majorly all regions in Ethiopia get annual rains in the summer season (the wet season)(June, July and august). All other seasons are almost dry season [53]**.**

### Covariates

Based on the previous research findings, the following covariates were selected: cigarette smoking history of the mother, father's education status, household wealth index, anemia level of the mother, women occupation type, residence, source of drinking water, type of toilet facility, mothers weight, mothers height, antenatal care visit history and birth interval.

### Statistical analysis

SPSS version 20 was used to analyze the data. Descriptive statistics was used to present the study variables. Principal component analysis was used to construct a household wealth index from the total assets in the household. Binary and multivariable logistic regression models were employed to determine the association between birth season and stunting. Crude odds ratios (COR) and Adjusted Odds Ratios (AOR) were presented with 95% confidence intervals. Each covariate was included in the multivariable logistic model regardless of their statistical significance in the binary logistic regression analysis. Finally statistical significance was declared at *p*-value < 0.05 [54].

## Result

The current study assessed the effect of birth season on chronic malnutrition among children underfive yearsin Ethiopia. We included 8855 children under five years from EDHS 2016 dataset. Almost fifty percent (51%) of children under-five years were male in sex and 89.1% of children resided in rural Ethiopia. Seventy-four percent of children’s mothers had antenatal care visits and eight-nine percent of children utilized unimproved toilet type (Table 1).

**Table 1:** Sociodemographic and behavioral characteristics of study participants (*N* = 8855)

Category		Frequency	Percent (%)
Sex of child	Male	4511	51
	Female	4344	49
Residence	Rural	7229	89.1
	Urban	1626	10.9
Father educational level	No education	5645	65.6
	Primary	2283	27.3
	Secondary	609	4.8
	Higher education	318	2.3
Household wealth index	Poor	4727	46.6
	Middle	1274	21
	Rich	2854	32.5
Anemia level of the mother	Severe	140	1.4
	Moderate	837	6.4
	Mild	2019	22.1
	Not anemic	5630	70.1
Mothers occupation type	Not working	5189	55.2
	Manual work	418	4.5
	Professional	1133	19.3
	Agricultural	1936	24.2
Antenatal care visit history	No	2050	25.7
	Yes	6791	74.2
Source of drinking water	Improved	5291	55.4
	Unimproved	3480	43.4
	Not adejure resident	84	1.2
Household toilet type	Improved	1481	9.7
	Unimproved	7290	89.1
	Not a dejure resident	84	1.2
Birth interval	< 24 months	1660	16.8
	24–47 months	3707	43.6
	48 months and above	3488	39.6
Cigarette Smoking history of the mother	No	8772	99.3
	Yes	83	0.7

### Prevalence of stunting in children under-five years

**Fig. 2:**
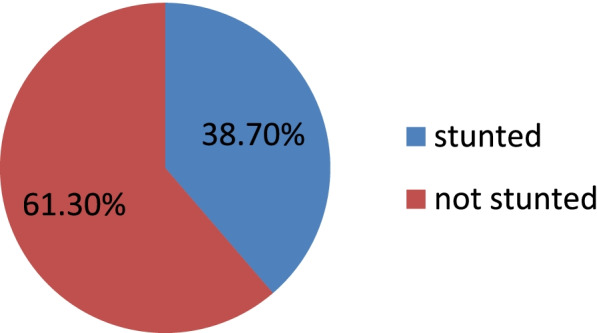
Stunting prevalence in children under five years

The prevalence of stunting among children under five years was 38.7% (1650) (95% CI: 36.8, 40.6) (Fig. 2).The birth season disaggregated prevalence of stunting was 25.2% in winter,27.7% in spring,25.7% in summer and 21.4% in autumn.

### The association between birth season and stunting (Chronic malnutrition)

In the multivariable logistic regression model, twelve variables were included to control the confounding effect. The birth season had a significant association with stunting both in the binary and multivariable logistic regression model. The odds of being stunted among children born in the spring season were decreased by 16% as compared to children born in the winter season (Table 2).

**Table 2:** Binary and multivariable logistic regression analysis of birth season and stunting in Ethiopia

		Stunting	
Variable		Unadjusted	Adjusted
	Category	COR: 95% CI	AOR:95% CI
Birth Season	Winter	Ref	Ref
	Spring	0.84 (0.7,0.9)	0.84:( 0.7,0.9)
	Summer	0.88:(0.7,1.1)	0.87 (0.7,1.1)
	Autumn	0.93: (0.7,1.2)	0.93 (0.7,1.2)

Adjusted variables: Region, cigarette smoking history of mothers,father education status, Household wealth index, anemia level of the mothers, women occupation type, residence, source of drinking water, type of toilet facility, mothers weight, mothers height, antenatal care visit and birth interval

## Discussion

The current study assess the association between birth season and chronic malnutrition (Stunting) extracted from EDHS 2016. The study concluded that there is a significant correlation between birth season and stunting. This is the first study that explored the association between birth season and stunting at the national level in Ethiopia.

Being born in the spring season reduced the occurrence of stunting as compared to children born in the winter season. The current finding is consistent with a study conducted in Ethiopia that assured the weight gain of an infant was high in the spring season [55]. Similarly infants born in the spring season had high velocities in length and weight catch up [56].Another study conducted in Southwest Ethiopia among children briefly shown that the mean weight and height gain was high in spring season [41].

The birth season effect is not only in children anthropometry although it affects the blood cell profiles.As reported in a study conducted in china the hemoglobin concentration was higher in winter-born infants as compared to summer-born infants [57].

Another study conducted in India confirmed that the birth size of offspring depends on the maternal intake of nutrients and physical activities during pregnancy. Both nutrient intake and physical activities of the mother determine the size of the offspring positively or negatively. For example, maternal activity and nutrient intake are high during harvest season for women who are participated in agricultural activity. Additionally, they also reported a winter-born infants had low anthropometric indices as compared to summer-born infants [58].High temperature during the intrauterine period and infancy downgrades the growth of an infant negatively and the consequences crossed to the adulthood period [59]. The birth season does not merely affect the nutritional status of the infant. It had also a significant effect on academic performance [60]. Some scholars correlate the season of birth with Vitamin –D exposure [61]. Moreover; the temperature level at the time of birth season affects the infants' anthropometry due to disruption of Vitamin D synthesis [62].Further more birth season had a significant correlation with cardiovascular, mental, and neurovascular diseases [63, 64].

It is possible to conclude that birth season had a significant impact on children's nutrition profiles based on the aforementioned literatures. The possible reason for the association between spring season and stunting is described as follows; In Ethiopia's context the spring season (also known as harvest season) had a surplus production and availability of fruit and vegetables in the garden, the markets and on the table. Though this physical accessibility of fruit and vegetables increased the chance of consumption for the women at (pregnancy or lactating period). Most fruit and vegetables become fruitful in the spring season. As a result, the women will get an adequate amount of vitamins and minerals; which is crucial for the child's growth and development. Seasonal hunger is common, in most of the country territory summer season is the worst (most households are food insecure) [65, 66].

Children born in hunger season are highly malnourished [67] and food insecured [68]. Empierically it is proved that stunting prevalence was varied in different season as reported from study conducted in Southern Ethiopia [65, 69]. A nationawide study conducted on daily energy intake and household dietary diversity score across months, sepctulate the energy intake and household dietary diversity score incresead from september to march in rural Ethiopia [51]. This research clearly depicted the dietary intake up and downs across season in Ethiopia. Energy intake and household dietary diversiity was inadequate in summer season( June to September).

Climate change is a big deal for global under nutrition especically the figure of undernourished people will increase in non determined folds in the future [70]. Thus climate change affect the nature trends of seasons.

As the second point; women who gave birth in spring will get adequate rest to give care to the child; it is well known that the next consecutive six months after spring season were low workload in rural Ethiopia. Additionally, during the consecutive six months after spring is relatively Ethiopian households were food secured.

In new point of view; malnutrition and disease depend on the lunar cycle and astronomical body movement.Recently different scholars hypothesized that human health,nutrition and solar system are interwoven.So our solar system (the universise) affect our daietary habit and it affect our nutritional status.Indeed day-night length was difreent across seasons;this probably affect the child physiology (growth and development). Actually this astrological effect is not well understood and it needs further investigation [71–73].

This research has a number of strengths but it has some limitation for example there is lack of researches in the area conducted before which made the discussion too difficult.

## Conclusion

Children born in spring season were less likely to be stunted; the researcher arbitrary called October effect. The clear scientific relation between birth season and child anthropometry is not well understood. For future researcher conducting follow up study and assessing the effect of birth season on childhood nutritional status will be a big assignment.

## Data Availability

The data was obtained from the website https://www.idhsdata.org/idhs/news.shtml.
